# Clinical significance of different *Histoplasma* genotypes in human histoplasmosis: insights from a multicenter study from Québec comparing North American (NAm2) with Latin American (LAmA) isolates

**DOI:** 10.1128/jcm.00551-26

**Published:** 2026-10-27

**Authors:** Jordan K. Mah, Jean-Daniel Talbot, Lucila Baldassarre, Soumya S. Moonjely, Donald C. Vinh, René Pelletier, Christian Lavallée, Cécile Tremblay, Eric Fournier, Philippe J. Dufresne, Simon F. Dufresne

**Affiliations:** 1 Department of Medicine, Hôpital Maisonneuve-Rosemont, CIUSSS-de-l’Est-de-l’Île-de-Montréal 60301, Montreal, Québec, Canada; 2 Centre de Recherche de l’Hôpital Maisonneuve-Rosemont, Montréal, Québec, Canada; 3 Department of Microbiology, Infectious Diseases and Immunology, Faculty of Medicine, Université de Montréal, Montréal, Québec, Canada; 4 Département de médecine spécialisée, Centre intégré de santé et de services sociaux des Laurentides 113711, Saint-Jérôme, Québec, Canada; 5 Laboratoire de santé publique du Québec, Institut national de santé publique du Québec 54470, Sainte-Anne-de-Bellevue, Québec, Canada; 6 Centre universitaire de santé McGill 5620 , Montréal, Québec, Canada; 7 Centre Hospitalier Universitaire de Québec – Université Laval 36896, Québec City, Québec, Canada; 8 Centre hospitalier de l'Université de Montréal, Montréal, Québec, Canada; University of Utah Health Medicine, Salt Lake City, Utah, USA

**Keywords:** Histoplasmosis, molecular genotyping, fungal infections, endemic mycosis, dimorphic fungi, NAm2, LAmA, *Histoplasma ohiense*, *Histoplasma capsulatum*, *Histoplasma suramericanum*

## Abstract

Histoplasmosis is recognized as endemic in Québec; however, no molecular epidemiological studies have been conducted to date. The clinical significance of identifying *Histoplasma capsulatum* genotypes remains unclear. We performed a retrospective multicenter study of 67 culture-confirmed histoplasmosis cases from four major academic hospitals in Québec between 1988 and 2014. Multi-locus sequence typing (MLST) was performed on isolates, and clinical data were analyzed for genotype-specific associations with demographics, clinical presentations, and outcomes. Most isolates belonged to either the North American clade 2 (48.1%) or Latin American clade A (39.2%), highlighting both endemic and imported disease patterns in Québec. A novel NAm2 sequence type (ST81) was identified, which was unique to Québec and accounted for 61% of the NAm2 isolates, but was not associated with significantly different clinical outcomes compared with other NAm2 strains. In multivariate analysis, disseminated disease was independently associated with immunosuppression (OR 14.18, *P* = 0.001), male sex (OR 6.01, *P* = 0.035), and LAmA genotype (OR 5.02, *P* = 0.031). Severe outcomes (intensive care unit admission, intubation, death, or amphotericin B use) were associated with immunosuppression (OR 4.48, *P* = 0.023), with a non-significant trend with LAmA infections (OR 3.06, *P* = 0.069). This first molecular characterization of histoplasmosis infections in Québec suggests a dual epidemiology, with locally acquired NAm2 and LAmA infections more often associated with travel or origin from endemic regions outside Québec. Host factors were associated with disease severity, while the potential contribution of fungal genotype requires further study. These findings highlight the value of molecular characterization for understanding the epidemiology of histoplasmosis in endemic settings.

**IMPORTANCE:**

Histoplasmosis is an important dimorphic fungal infection in North America. Although Québec is a recognized endemic region, its molecular epidemiology has not been previously characterized, and the clinical relevance of different *Histoplasma* genotypes remains uncertain. In this multicenter study, we performed multi-locus sequence typing with detailed clinical data from 67 culture-confirmed cases diagnosed between 1988 and 2014. This represents the first molecular characterization of clinical *Histoplasma* isolates from Québec and the largest Canadian cohort examining the association between molecular genotypes, clinical presentation, and outcomes. We identified both North American NAm2 (*Histoplasma ohiense*) and imported Latin American LAmA (*Histoplasma suramericanum*) lineages, suggestive of a combination of locally acquired and travel-associated infections. We also identified a novel NAm2 sequence type (ST81) that appears unique to Québec, suggesting possible regional circulation. The LAmA genotype was associated with disseminated histoplasmosis, whereas immunosuppression was the principal predictor of severe outcomes. Overall, host factors were associated with disease severity, while the potential contribution of fungal genotype warrants further study. These findings highlight the value of molecular characterization for understanding the epidemiology of histoplasmosis in endemic settings.

## INTRODUCTION

Histoplasmosis is a potentially life-threatening fungal infection caused by *Histoplasma* spp., a dimorphic fungus that thrives in nitrogen-rich soils, particularly those contaminated by bird or bat droppings (1). Although often asymptomatic in immunocompetent individuals, histoplasmosis may cause pulmonary disease that can progress to chronic or disseminated infection, particularly following high-inoculum exposures or among persons with comorbidities or immunosuppression (1, 2).

Historically considered endemic to select regions, *Histoplasma* spp. are now recognized as having a broader, worldwide distribution, with hyperendemic areas in the Americas, Africa, and parts of Asia (3). Recent studies in the United States also indicate broader distribution and higher incidence in some regions not previously considered endemic (4–6). In Canada, Québec and southeastern Ontario, particularly along the St. Lawrence River, have long been recognized as endemic areas (7). More recently, cases have also emerged in western Canada, suggesting a wider ecological niche than previously appreciated (8).

Molecular studies have revealed that *Histoplasma* represents a species complex with several genetically distinct phylogenetic clades, some of which have recently been assigned formal species names, including NAm1 (*Histoplasma mississipiense*), NAm2 (*Histoplasma ohiense*)*,* and LAmA (*Histoplasma suramericanum)* (9–12). These clades have distinct geographic distributions; NAm1 and NAm2 predominate in North America, while LAmA is more commonly reported in Latin America (10). Emerging evidence suggests potential differences in virulence and clinical manifestations between genotypes, such as varying severity and tissue tropism, although data remain limited, and the clinical relevance of these distinctions is not fully understood (12–16).

Despite its recognized endemicity and recent emergence, molecular epidemiological studies on histoplasmosis in Canada are limited (8). Québec has long been recognized as an endemic region; however, no genomic studies have yet characterized the local molecular epidemiology of *H. capsulatum* nor distinguished locally acquired from imported cases of infection. In this study, we aimed to describe the genotypic diversity of *H. capsulatum* in Québec and assess the potential associations between specific genotypes and patient characteristics and clinical outcomes.

## MATERIALS AND METHODS

### Study design

This was a retrospective, multicenter descriptive study across four of Québec’s major academic hospitals. Histoplasmosis cases were identified based on positive cultures received and conserved by the Laboratoire de santé publique du Québec (LSPQ), a centralized provincial laboratory of Québec, from 1988 to 2014. As *H. capsulatum* is a risk group 3 organism in Canada, a provincial directive requests that all clinical isolates from the province be referred to LSPQ for confirmation of identification.

Four major hospitals across Québec were selected based on accessibility and feasibility for clinical data collection. We aimed to sequence approximately 90 isolates, representing approximately 60% of the culture-confirmed histoplasmosis cases in Québec. Clinical isolates were sequenced using multi-locus sequence typing (MLST) and classified into genotypes by phylogenetic analysis. Clinical outcomes and demographic characteristics were analyzed according to genotype.

### Data collection

Basic demographic and specimen information (age, sex, region of residence, health region, hospital, and sample type) were obtained from the LSPQ laboratory information system. Investigators at each site performed detailed chart reviews using a standardized case report form (J.T. and S.D., Hôpital Maisonneuve-Rosemont; J.T. and D.V., Centre universitaire de santé McGill; R.P., Centre hospitalier universitaire de Québec; and J.T. and C.T., Centre hospitalier de l'Université de Montréal). Collected data included demographics (age, sex, country of origin, and ethnicity), comorbidities, immunosuppressive status and therapies, exposure risks, travels, clinical presentation, laboratory findings, imaging, antifungal therapy, complications, and outcomes.

### Case definitions

Patients were categorized into invasive fungal disease by the 2020 European Organization for Research and Treatment of Cancer and the Mycoses Study Group Education and Research Consortium (EORTC/MSGERC) (17). Clinical syndrome was categorized based on the clinical manifestations outlined by the Infectious Diseases Society of America (IDSA) clinical practice guidelines for histoplasmosis (18). Determination of travel to endemic areas for histoplasmosis outside of Québec was based on the known areas of endemicity and geographic distributions (3).

Older age was defined as ≥55 years. Two study investigators (J.T. and S.D.) determined histoplasmosis-attributable mortality, defined as any death occurring during targeted antifungal therapy or within 30 days of its discontinuation, in which histoplasmosis was judged to be the primary or a major contributing cause of death (progressive fungal disease or organ failure in the setting of active histoplasmosis). Severe clinical outcomes included any one of the following: intensive care unit (ICU) admission, endotracheal intubation, death, or amphotericin B treatment. Organ involvement definitions for disseminated disease were based on a combination of clinical, laboratory, or microbiological/histopathological criteria (Supplemental material).

### Molecular methods and genotyping

MLST was performed at LSPQ with methods as previously described (10, 19). Clinical isolates were heat-killed (100°C for 15 min), and DNA was extracted using a cetyltrimethylammonium bromide (CTAB) chloroform-isoamyl alcohol protocol. Four partial protein-coding genes (*Arf*, ADP-ribosylation factor; *H-anti*, H-antigen precursor; *Ole*, delta-9 fatty acid desaturase; and *tub1*, alpha tubulin) were amplified via polymerase chain reaction (PCR), purified, and sequenced using the Sanger method to detect genetic polymorphisms.

Sequences were compared to other *Histoplasma* sequences in the NCBI database using BLAST and aligned with 80 reference sequences via BioNumerics (v7.0). A phylogenetic tree was generated using neighbor-joining and maximum parsimony methods, applying Kimura’s two-parameter model with 1,000 bootstrap replications.

Isolates were classified into seven phylogenetic groups as identified by prior studies (10, 19). The concatenated data set (1,585 bases) was retrieved from TreeBASE (study ID S1063, Dataset S1), and the matrix was separated by gene according to the length and order reported by the Kasuga et al. (19). The Eurasian clade was classified within the LAmA group given more recent phylogenetic analyses, suggesting that this is not a distinct species and that it originated from a branch of the LAmA clade (10).

### Comparative genotype validation

To assess if Québec *Histoplasma* isolates were genetically distinct, the genotypes for *Ole*, *Arf,* and *H-anti* were compared to those of Quan et al. (11) and Dingle et al. (8), with 491 and 11 isolates, respectively. The latter 11 isolates, recovered from patients in Alberta, are, to our knowledge, the only genotypically characterized *Histoplasma* isolates previously described from Canada (8). *Ole, Arf,* and *H-anti* regions were extracted from whole-genome sequencing (WGS) assemblies with the NCBI toolkit if needed, and the three genes were concatenated. Across the 675 isolates, the mean concatenated sequence length was 1,290 nucleotides. An initial raw alignment was performed with MAFFT v.7.4 (20) and manually edited in MEGA 11 (21) to remove sites containing large gaps (> 30 nucleotides). A second alignment on the final 952 conserved nucleotide site data set was produced using the maximum likelihood algorithm on the multiple alignment generated with IQ-TREE v2.2.0 with the ModelFinder Plus (MFP) option and node support verified with 500 non-parametric bootstrap replicates. Sequences were deposited in GenBank under a BioProject accession (ID: PRJNA1393768).

### Microbiological and clinical outcome analysis

Clinical and demographic characteristics were compared across genotypes. Disseminated histoplasmosis (DH) cases were analyzed by organ involvement. Risk factors for DH and severe outcomes were assessed using univariate and multivariate binary logistic regression.

### Data analyses

Continuous data were summarized as means with standard deviation (SD) and compared using a *t*-test. Categorical data were compared using the Fisher exact test or χ² test, while continuous variables were compared using a *t*-test or ANOVA. A *P*-value of <0.05 was considered statistically significant. SPSS version 28 (IBM, Chicago, Illinois) was used for analysis.

### Regression analysis

Univariate and multivariate logistic regression models were used to identify the predictors of disseminated disease and severe outcomes. Odds ratios (OR) and 95% confidence intervals (CI) were reported. Reference categories were based on the largest or most typical population groups. These analyses were limited to the two predominant genotypes (NAm2 and LAmA) because of the small number and heterogeneity of other genotypes. Variables were selected based on clinical relevance, biological plausibility, and previously reported associations in the literature.

## RESULTS

### Case identification

**Fig 1:**
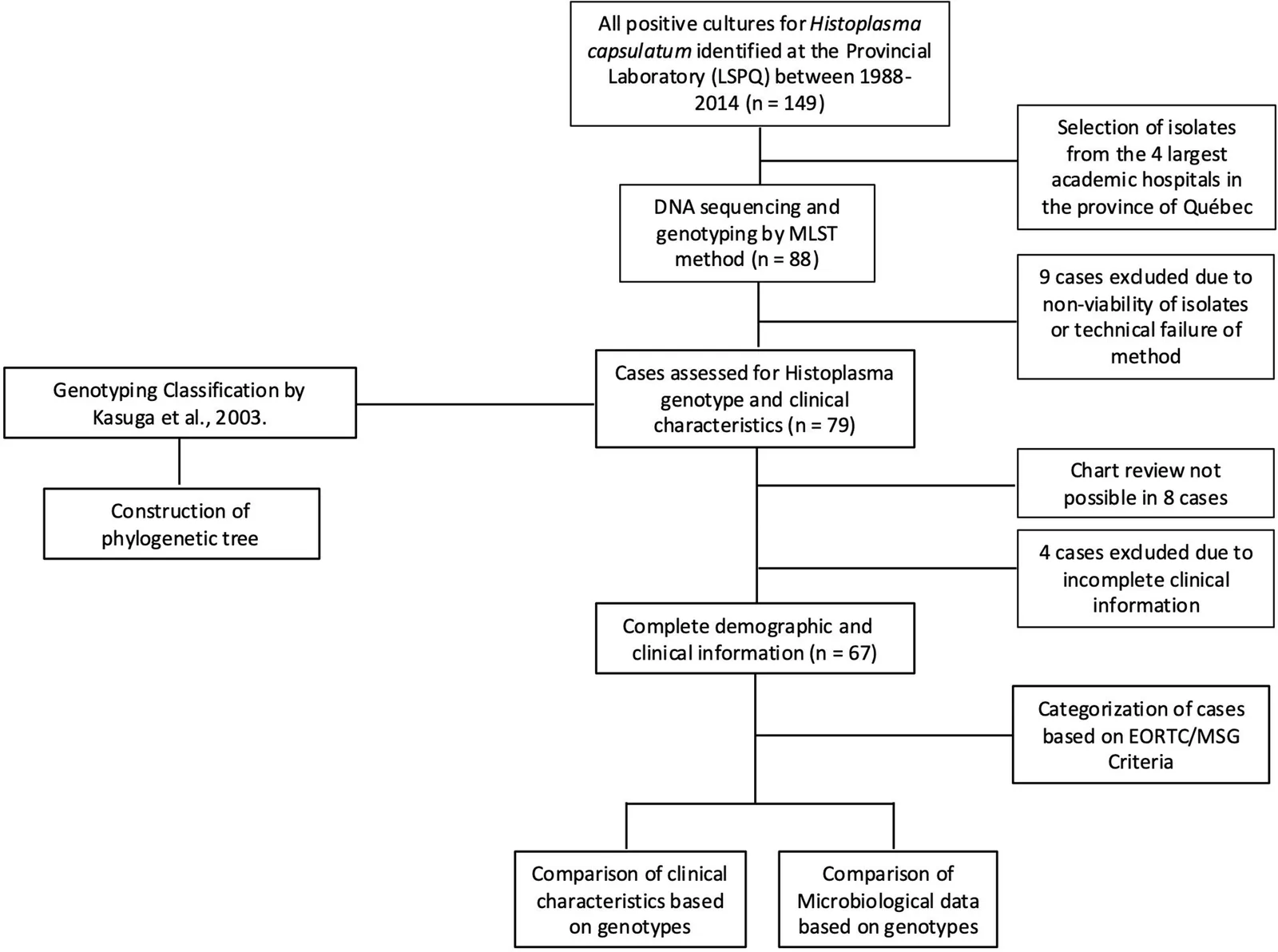
Study design. EORTC/MSG, European Organization for Research and Treatment of Cancer and the Mycoses Study Group Education and Research Consortium; MLST, Multi-locus sequence typing; LSPQ, Laboratoire de santé publique du Québec.

A total of 149 culture-positive *H. capsulatum* isolates were available in the LSPQ biobank. Of these, 88 originated from the four selected hospitals. Seventy-nine isolates were successfully sequenced and included for molecular epidemiology analyses. These isolates were then matched with clinical records; ultimately, 67 cases had complete demographic and clinical information and constituted the main cohort included in the final analysis (Fig. 1).

### Phylogenetic tree

**Fig 2:**
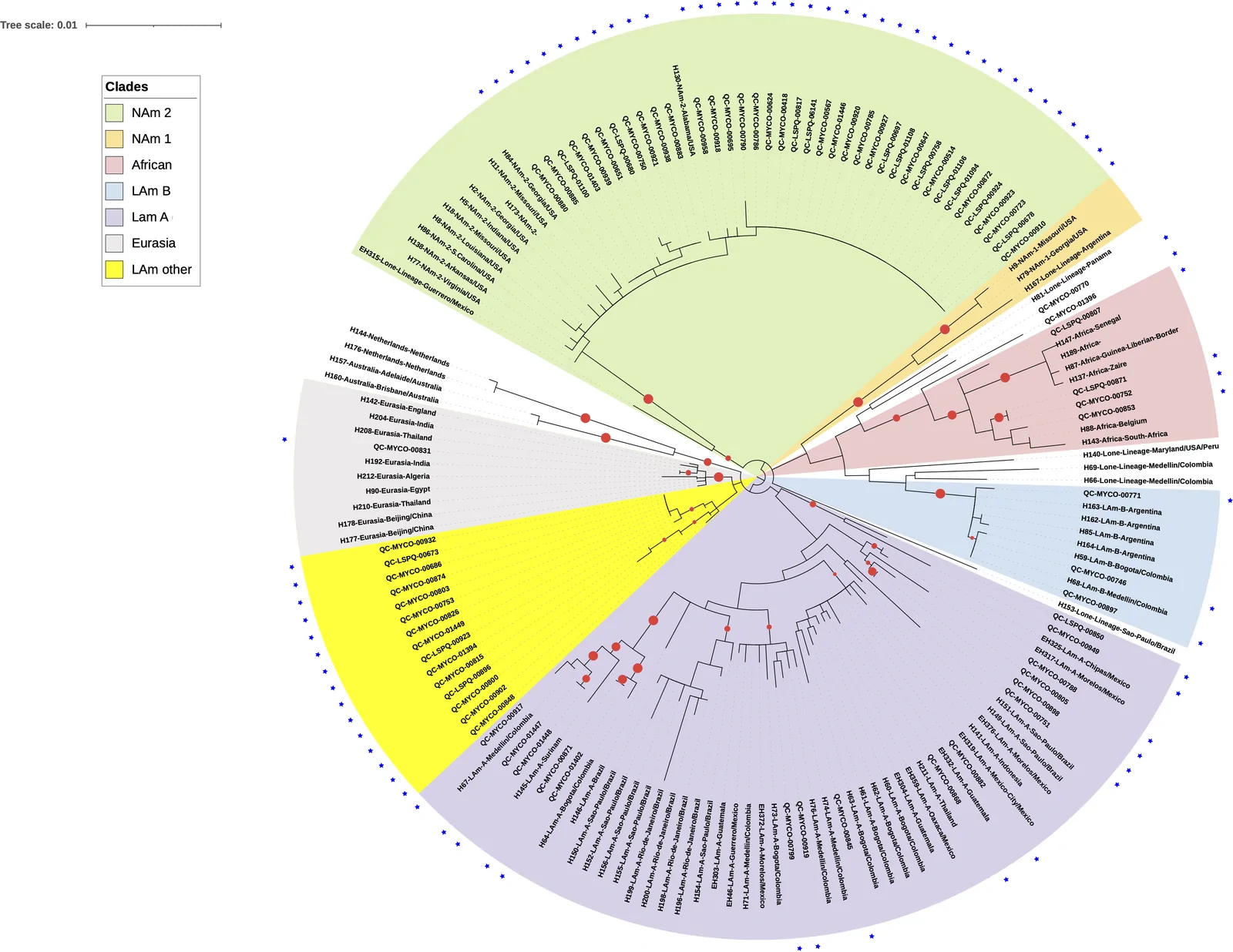
Maximum parsimony phylogenetic tree of clinical strains of *Histoplasma* spp. according to genotype, aligned with 80 reference sequences. In the Latin American group, this was further divided into LAmA, LAmB, and LAm other, based on new clades proposed (group D, group E, and Rio de Janeiro [RJ]). Blue asterisks represent Québec strains from our study. NAm2, North American clade 2; NAm1, North American clade; LAmA, Latin American clade A; LAmB, Latin American clade B; and LAm other, Latin American other than A/B.

Phylogenetic analysis of 79 isolates was based on *Ole*, *Arf*, and *H-anti* regions aligned with 80 reference sequences (Fig. 2). There were 38 (48.1%) NAm2, 31 (39.2%) LAmA (including the LAm other group), 4 (5.1%) African, 3 (3.8%) LAm B, 1 (1.3%) Eurasia, 2 (2.5%) lone lineage, and 0 NAm1. The LAm other group was previously classified within the LAmA group (10), but new clades/cryptic species within this group have been proposed (11).

### Baseline patient characteristics, clinical presentation, and outcomes

Among the 67 cases, 70.1% (47/67) were male, with a mean age of 46.3 ± 14.9 years. The majority of patients in the study were Caucasian (76.1%, 51/67), followed by Hispanic (10.4%, 7/67), while other ethnicities represented 13.4% (9/67). Overall, 49 (73.1%) patients were immunosuppressed. There were 43.3% (29/67) patients with human immunodeficiency virus (HIV); 93.1% (27/29) met criteria for acquired immunodeficiency syndrome (AIDS), with a mean CD4 count of 32.2 ± 37.0. Additionally, immunosuppression unrelated to HIV was present in 29.9% (20/67), including solid organ transplant (11.9%, 8/67), rheumatological disease (7.5%, 5/67), or hematological malignancy (3.0%, 2/67). Baseline characteristics are summarized in Table 1.

**TABLE 1:** Baseline characteristics and clinical outcomes^*a*^^,^^*b*^^,*c*^

Characteristics	All patients (*n* = 67)
Age	46.3 ± 14.9
Male sex	47 (70.1)
Ethnicity	
Caucasian	51 (76.1)
Hispanic	7 (10.4)
Other	9 (13.4)
Place of birth	
North America	50 (74.6)
Central America	7 (10.4)
South America	3 (4.5)
Europe	2 (3.0)
Africa	4 (6.0)
Unknown	1 (1.5)
Reported history of travel outside of Québec to an endemic area	28 (41.8)
Comorbidities	
Diabetes	8 (11.9)
COPD	10 (14.9)
Immunosuppression	49 (73.1)
HIV	29 (43.3)
AIDS	27 (93.1)
Average CD4 count	32.2 ± 37.0
Non-HIV	20 (29.9)
Hematological malignancy	2 (3.0)
Solid organ transplantation	8 (11.9)
Rheumatological disease	5 (7.5)
Other	5 (7.5)
Clinical syndrome	
Acute PH	15 (22.3)
Chronic PH	10 (14.9)
Disseminated histoplasmosis	42 (62.7)
Other	0 (0)
Classification based on EORTC/MSG criteria	
Proven histoplasmosis infection	67 (100.0)
Average duration of hospitalization (in days)	32.8 ± 32.2
Endotracheal intubation or admission to ICU	10 (14.9)
Mortality^*c*^	11 (16.4)
Treatment with amphotericin B	40 (59.7)

^*a*^ COPD, chronic obstructive pulmonary disease; EORTC/MSG, European Organization for Research and Treatment of Cancer and the Mycoses Study Group Education and Research Consortium; HIV, human immunodeficiency virus; ICU, intensive care unit; PH, pulmonary histoplasmosis; AIDS, acquired immunodeficiency syndrome.

^*b*^ Percentage is represented in parentheses, and ± correspond to the standard deviation.

^*c*^ Represents mortality attributable to *Histoplasma* infection.

All cases were proven *Histoplasma* infection based on EORTC/MSG criteria. The most common presentation was DH (62.7%, 42/67), followed by acute pulmonary histoplasmosis (22.3%, 15/67). There were only 10 (14.9%, 10/67) cases of chronic pulmonary histoplasmosis. The average duration of hospitalization was 32.8 days (± 32.2). Among the 67 patients, 14.9% (10/67) required endotracheal intubation or ICU admission, and 59.7% (40/67) required treatment with Amphotericin B. The mortality attributable to *Histoplasma* infection during the study was 16.4% (11/67).

### Genotyping distribution and molecular findings

Among the *Histoplasma* isolates with complete demographic and clinical information, 49.2% (33/67) belonged to the NAm2 clade, followed by 41.8% (28/67) classified as LAmA. There was also 9.0% (6/67) from other genotypes, including 4.5% (3/67) African, 1.5% (1/67) from LAmB, and 3.0% (2/67) from lone lineages (Fig. 2). Within the NAm2 group, there was a unique sequence type that emerged, sequence type 81 (ST81), which had not been described previously (BioProject ID: PRJNA1393768). This sequence type corresponded to 60.6% (20/33) of NAm2 cases. ST81 comparison against an exhaustive international survey of *Histoplasma* genotypes (11) and of Alberta, Canada (Dingle TC and Croxen MA), did not match any previously described isolate. This expanded IQ-TREE analysis was performed as a comparative validation of the Québec ST81 lineage within a larger contemporary global Histoplasma data set, rather than as a separate classification framework. Both phylogenetic approaches yielded concordant results.

### Clinical outcomes and disease severity by genotype

We compared the clinical characteristics and outcomes between ST81 and other sequence types within the NAm2 group. There were no significant differences between the two groups with respect to DH (45.0% [9/20] vs 46.2% [6/13], *P* = 0.95) or mortality (20% [4/20] vs 15.4% [2/13], *P* = 0.74).

When comparing LAmA to NAm2, a significantly higher proportion of LAmA cases had a history of travel to histoplasmosis endemic areas outside of Québec (67.9% [19/28] vs 24.2% [8/33], *P* = 0.0006). In contrast, NAm2 more often had an infecting genotype that was concordant with the patient’s reported place of birth (97.0% [32/33] vs 25.0% [7/28], *P* = 0.000000001) (Table 2). LAmA infections were also associated with a higher rate of DH compared to NAm2 (75% [21/28] vs 45.5% [15/33], *P* < 0.02). There were no significant differences in overall immunosuppression, duration of hospitalization, or mortality; however, the types of immunosuppression varied within each genotype. HIV infection was significantly more frequent in the LAmA group than in the NAm2 group (64.3% [18/28] vs 21.2% [7/33], *P* = 0.0006), and among patients with DH (85.7% [18/21] vs 46.7% [7/15], *P* = 0.012). Conversely, non-HIV immunosuppression was more common in the NAm2 group, both overall (42.4% [14/33] vs 17.9% [5/28], *P* = 0.0394) and among patients with DH (53.3% [8/15] vs 14.3% [3/21], *P* = 0.012). A summary of these results is displayed in Table 2.

**TABLE 2:** Comparison of demographics, clinical outcomes, and disease severity based on genotype^*b*^

	NAm2 (*n* = 33)	LAmA (*n* = 28)	Other (*n* = 6)
Place of Birth			
North America	32 (97.0)	18 (64.3)	0
Central America	0	7 (25.0)	0
South America	0	1 (3.6)	2 (33.3)
Europe	1 (3.0)	1 (3.6)	0
Africa	0	1 (3.6)	3 (50.0)
Unknown	0	0	1 (16.7)
Genotype – birthplace concordance	32 (97.0^*a*^)	7 (25.0)^*a*^	3 (50.0)
Reported history of travel outside of Québec to an endemic area	8 (24.2)^*a*^	19 (67.9)^*a*^	1 (16.7)
Clinical syndrome			
Acute PH	10 (30.3)	5 (17.9)	0
Chronic PH	8 (24.2)	2 (7.1)	0
DH	15 (45.5)^*a*^	21 (75.0)^*a*^	6 (100)
Other	0	0	0
Immunosuppression	21 (63.6)	23 (82.1)	5 (83.3)
HIV/AIDS	7 (21.2)^*a*^	18 (64.3)^*a*^	4 (66.7)
Other	14 (42.4)^*a*^	5 (17.9)^*a*^	1 (16.7)
Average duration of hospitalization	36.2 ± 34.9	28.9 ± 28.8	28.8 ± 16.5
Hospitalization ≥ 7 days	27 (81.8)	24 (85.7)	4 (66.7)
Hospitalization ≥ 14 days	21 (63.6)	21 (75.0)	4 (66.7)
Hospitalization ≥ 28 days	9 (27.3)	13 (46.4)	2 (33.3)
Endotracheal intubation or admission to ICU	3 (9.1)	6 (21.4)	1 (16.7)
Mortality	6 (18.2)	4 (14.2)	1 (16.7)
Treatment with amphotericin B	15 (45.5)^*a*^	20 (71.4)^*a*^	5 (83.3)

^*a*^ Indicates significant differences between groups.

^*b*^ PH, pulmonary histoplasmosis; DH, disseminated histoplasmosis; ICU, intensive care unit; NAm2, North American clade 2; LAmA, Latin American clade A; HIV, human immunodeficiency virus; AIDS, acquired immunodeficiency syndrome. Percentages are represented in parentheses, while ± correspond to the standard deviation.

**Fig 3:**
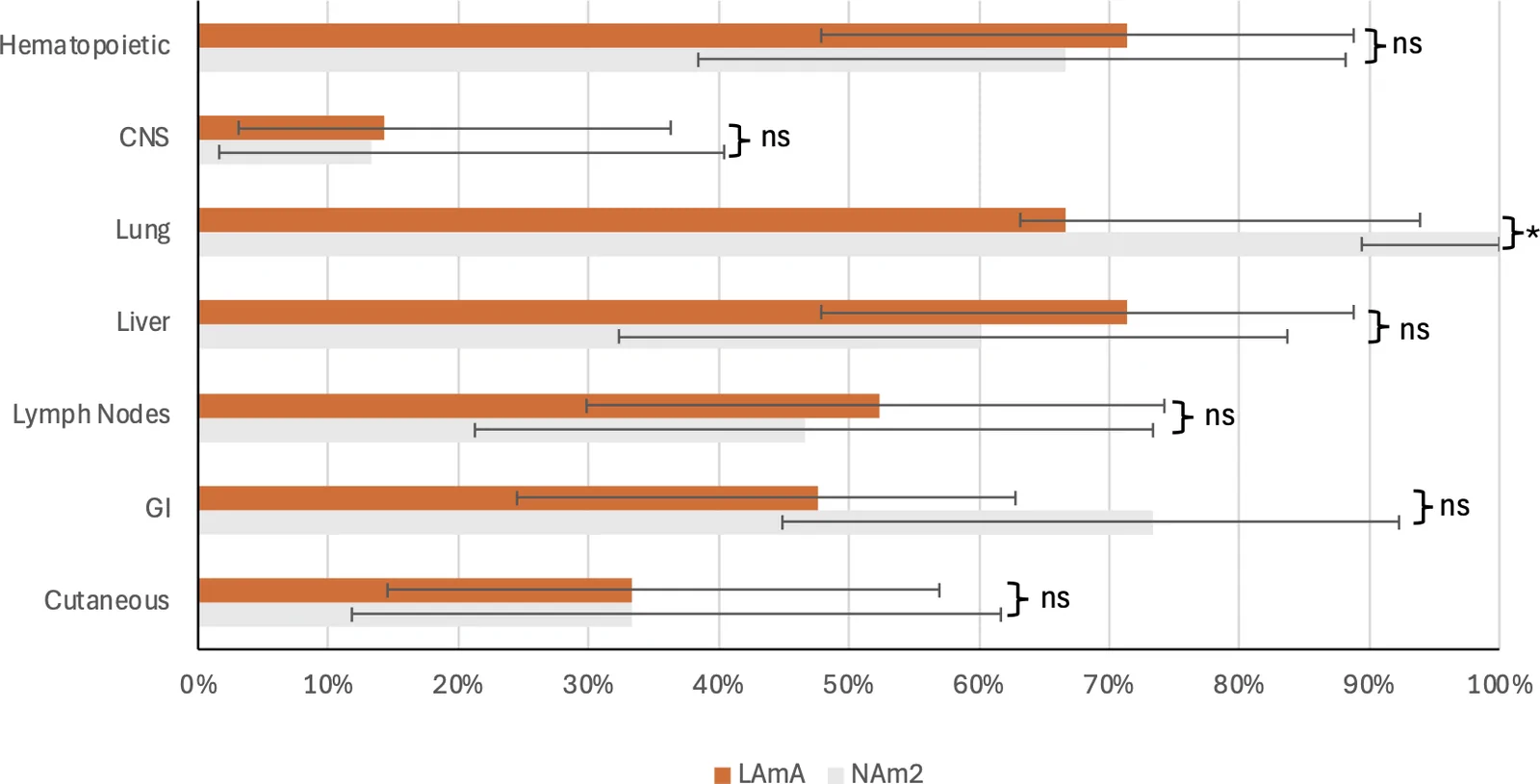
Organ systems affected in disseminated histoplasmosis, according to genotype.* = statistical significance; ns = no statistical significance. NAm2, North American clade 2; LAmA, Latin American clade A; CNS, central nervous system; GI, gastrointestinal.

In cases of disseminated histoplasmosis, the distribution of organ systems affected by genotype is shown in Fig. 3. Lung involvement was more frequent among NAm2 infections (100% [15/15] vs 66.7% [14/21], *P* = 0.027), whereas the frequencies of other organ involvements did not differ significantly between genotypes.

### Predictors of disseminated disease and severe clinical outcomes

By univariate analysis, male sex, presence of immunosuppression, and infection with the LAmA genotype were each associated with higher odds of disseminated disease. In multivariate analysis, these associations were statistically significant with an OR of 6.01 (95% CI 1.14–31.68, *P* = 0.035), 14.18 (95% CI 2.90–69.23, *P* < 0.001), and 5.02 (95% CI 1.16–21.64, *P* = 0.031), respectively. Older age was not associated with an increased risk of disseminated disease (Table 3).

**TABLE 3:** Univariate and multivariate analysis for risk factors for disseminated disease^*a*^^,^^*b*^

Potential risk factor or exposure	Proportion with disseminated disease (%)	Univariate OR (95% CI)	*P*-value	Multivariate OR (95% CI)	*P*-value
Age					
<55	26/44 (59.0)	1.0 (Reference)	–	1.0 (Reference)	–
≥55	10/17 (58.8)	0.99 (0.32–3.09)	0.985	1.59 (0.35–7.21)	0.547
Gender					
Female	4/15 (26.7)	1.0 (Reference)	-	1.0 (Reference)	–
Male	**32/46 (69.6)**	**6.29 (1.70–23.19)**	**0.006**	**6.01 (1.14–31.68)**	**0.035**
Immunosuppression					
No	3/18 (16.7)	1.0 (Reference)	–	1.0 (Reference)	–
Yes	**33/43 (76.7)**	**16.5 (3.96–68.76)**	**<0.001**	**14.18** **(2.90–69.23)**	**0.001**
Genotype					
NAm2	15/33 (45.5)	1.0 (Reference)	–	1.0 (Reference)	–
LAmA	**21/28 (75.0)**	**3.60 (1.20–10.77)**	**0.022**	**5.02 (1.16–21.64)**	**0.031**

^*a*^ Analyses were restricted to NAm2 and LAmA genotypes (*n* = 61), while other genotypes (*n* = 6) were excluded due to small numbers and heterogeneity. Bold signifies statistical significance.

^*b*^ NAm2, North American Clade 2; LAmA, Latin American Clade A; CI, confidence interval, OR, odds ratio.

By univariate analysis, both immunosuppression and infection with the LAmA genotype were associated with higher odds of severe clinical outcomes. After adjustment in the multivariate model, only immunosuppression remained independently associated (OR of 4.48; 95% CI 1.23–16.35, *P* = 0.023). The LAmA genotype demonstrated a similar trend toward increased odds (OR 3.06, 95% CI 0.92–10.23, *P* = 0.069), although this did not reach statistical significance. Neither age nor gender was associated with higher odds of severe clinical outcomes (Table 4).

**TABLE 4:** Univariate and multivariate analysis for risk factors for severe clinical outcomes (endotracheal intubation, intensive care, treatment with amphotericin B, or death)^*a*^^,^^*b*^

Potential risk factor or exposure	Proportion with severe outcomes (%)	Univariate OR (95% CI)	*P*-value	Multivariate OR (95% CI)	*P*-value
Age					
<55	29/44 (65.9)	1.0 (Reference)	–	1.0 (Reference)	–
≥55	10/17 (58.8)	0.74 (0.23–2.33)	0.606	1.02 (0.29–3.64)	0.972
Gender					
Female	9/15 (60.0)	1.0 (Reference)	–	1.0 (Reference)	–
Male	30/46 (65.2)	1.25 (0.38–4.14)	0.715	1.29 (0.31–5.33)	0.721
Immunosuppression					
No	7/18 (38.9)	1.0 (Reference)	–	1.0 (Reference)	-
Yes	**32/43 (74.4)**	**4.57 (1.42–14.72)**	**0.011**	**4.48 (1.23–16.35)**	**0.023**
Genotype					
NAm2	17/33 (51.5)	1.0 (Reference)	–	1.0 (Reference)	–
LAmA	**22/28 (78.6)**	**3.45 (1.11–10.70)**	**0.032**	3.06 (0.92–10.23)	0.069

^*a*^ Analyses were restricted to NAm2 and LAmA genotypes (*n* = 61), while other genotypes (*n* = 6) were excluded due to small numbers and heterogeneity. Bold signifies statistical significance.

^*b*^ NAm2, North American Clade 2; LAmA, Latin American Clade A; CI, confidence interval; OR, odds ratio.

## DISCUSSION

This study provides the first molecular epidemiological characterization of clinical *Histoplasma* spp. from Québec and, to our knowledge, the largest Canadian cohort to link fungal genotypes with detailed clinical data (8). We observed differences in clinical presentations across *Histoplasma* genotypes, including variations in the proportion of patients with DH. These findings suggest that the pathogen genotype, alongside host factors, may influence the clinical presentation of histoplasmosis, although residual confounding by geography and host characteristics cannot be excluded.

Epidemiologically, the near-equal proportions of NAm2 (48.1%) and LAmA (39.2%) contrast with U.S. reports, where NAm2 (65%) and NAm1 (31%) genotypes predominate, while LAm genotypes (18%) are less frequent (22). The absence of NAm1 is distinct compared to other studies; however, because only a subset of provincial isolates was sequenced, NAm1 may be underrepresented in this cohort (22). In contrast, the predominance of NAm2 and LAmA may reflect Québec’s dual epidemiologic context, with NAm2 infections appearing more consistent with local acquisition and LAmA cases more frequently associated with immigration or travel from endemic regions. The identification of a novel NAm2 sequence type (ST81), unique to Québec, supports the presence of a regionally adapted, circulating strain, although this variant did not have any apparent clinical differences when compared with other NAm2 strains.

In multivariable models, infection with the LAmA genotype remained significantly associated with DH. This finding is consistent with experimental models, suggesting that LAmA isolates may induce more robust inflammatory responses, exhibit distinct histopathological patterns, and have been associated with higher mortality (13–16, 23). However, in our cohort, different genotypes were associated with distinct patterns of immunosuppression; LAmA infections occurred predominantly in people living with HIV/AIDS and were frequently linked to travel, whereas NAm2 infections were associated with non-HIV immunosuppression (transplant recipients and patients receiving immunomodulatory therapies) without a reported history of travel. As a result, it is difficult to disentangle the influence of fungal genotype from host characteristics (e.g., advanced HIV infection) and higher background exposure in hyperendemic settings. Alternative explanations, such as a greater burden of untreated HIV among migrants from Latin America and a higher incidence of histoplasmosis in that population, may partly account for the over-representation of LAmA-associated DH in our data. Future studies with larger, more diverse cohorts and more granular data on HIV stage, antiretroviral therapy use, and exposure history will be needed to clarify these relationships.

Beyond genotype, host factors also strongly influenced disease manifestations. Consistent with prior studies, immunosuppression was the strongest predictor of disseminated disease and severe outcomes (18). Male sex was also associated with disseminated disease, a finding that mirrors observations in coccidioidomycosis and may reflect sex-based differences in immune responses or behavioral exposure patterns (24–27).

Despite the higher dissemination rates observed with LAmA, mortality did not differ by genotype. This may reflect the influence of factors beyond fungal genotype, including timely diagnosis, access to liposomal amphotericin B, and the availability of intensive care in our setting. In addition, the lower overall mortality in our cohort (16.4%) compared with other series (21%–53%) may have reduced the power to detect modest differences in mortality between these two groups (28).

A key strength of this study is the inclusion of one of the largest cohorts of proven, culture-confirmed histoplasmosis cases with linked clinical and molecular data, enabling integrated genotype–phenotype analyses. Nevertheless, several limitations must be acknowledged. First, the retrospective study design and restriction to culture-positive cases from tertiary-care centers likely enriched the cohort for more severe disease and limited the ability to capture milder or subacute presentations diagnosed by non-culture-based methods. Second, the study period (1988–2014) spans major changes in HIV management, antifungal therapy, and supportive care; we were unable to fully adjust for these temporal trends, which could have influenced both dissemination rates and clinical outcomes. Third, our modest sample size may limit causal inference and was underpowered for subgroup analyses. For instance, we grouped different types of immunosuppression in regression models due to limited power in individual categories, which may obscure differences between HIV-related and non-HIV immunosuppression. Finally, although MLST is a validated genotyping tool for *Histoplasma*, it offers a lower resolution than WGS. While WGS has led to the reclassification of cryptic species within *H. capsulatum*, most isolates in our study belonged to LAmA or NAm2, groups not significantly impacted by taxonomic revision, supporting the robustness of our findings (9, 29–31). Moreover, MLST remains a practical and appropriate tool for molecular epidemiologic studies (11, 32).

In conclusion, this first molecular characterization of *Histoplasma* infections in Québec suggests the coexistence of NAm2 and LAmA strains, consistent with a dual epidemiological pattern involving both locally acquired and travel- or immigration-associated infections. Host factors were associated with disease severity, while the contribution of pathogen genotype warrants further study. These findings support the potential value of fungal genotyping, which is not currently part of routine diagnostics, to better understand the epidemiology of histoplasmosis, including the distinction between locally acquired and travel-associated disease, and to inform epidemiological surveillance as histoplasmosis is increasingly recognized in temperate regions and non-traditional areas.

## Data Availability

The MLST sequences generated in this study have been deposited in GenBank and are available under BioProject PRJNA1393768. The accession numbers are as follows: Arf, PX830495–PX830509, PX830511–PX830512, PX830514, and PX830517–PX830573; H-anti, PX797673–PX797687, PX797689–PX797690, PX797692, and PX797695–PX797751; Ole, PX797752–PX797766, PX797768–PX797769, PX797771, and PX797774–PX797830; and tub1, PX797831–PX797845, PX797847–PX797848, PX797850, and PX797853–PX797909.

## SUPPLEMENTAL MATERIAL

Supplemental material jcm.00551-26-s0001.docx Definitions for organ involvement were based on a combination of clinical, laboratory, or microbiological/histopathological criteria.

Dataset S1 jcm.00551-26-s0002.txt FASTA file of the 80 concatenated sequences.

ASM does not own the copyrights to Supplemental Material that may be linked to, or accessed through, an article. The authors have granted ASM a non-exclusive, world-wide license to publish the Supplemental Material files. Please contact the corresponding author directly for reuse.
