# Supplementary material for: Clinical significance of different Histoplasma genotypes in human histoplasmosis: insights from a multicenter study from Québec comparing North American (NAm2) with Latin American (LAmA) isolates
Source: J Clin Microbiol. 2026 Oct 27;64(11):e00551-26. doi: 10.1128/jcm.00551-26 (PMC13753795; doi:10.1128/jcm.00551-26)
Supplement: Supplementary file 1 [file jcm.00551-26-s0001.docx]

**Cutaneous:** rash compatible with histoplasmosis; skin biopsy positive; skin culture positive; positive histopathology.

**Gastrointestinal:** pharyngitis, esophagitis, gastritis, enteritis, colitis; hemoptysis, rectal bleeding (hematochezia), melena; biopsy culture positive; positive histopathology.

**Lymph nodes:** lymphadenopathy; generalized lymphadenopathy; splenomegaly; biopsy culture positive; positive histopathology.

**Liver:** hepatomegaly; ALT > 60 U/L; total bilirubin > 30 µmol/L; alkaline phosphatase > 200 U/L; biopsy positive; positive histopathology.

**Pulmonary:** compatible imaging (any nodule, infiltrate, or effusion); sputum culture positive; BAL positive; biopsy positive; positive histopathology.

**Central nervous system:** encephalitis, meningitis; CT scan with compatible lesions; CSF culture positive; positive histopathology; CSF serological testing positive.

**Hematological:** WBC < 4 × 10⁹/L; platelets < 140 × 10⁹/L; biopsy culture positive; positive histopathology.

Definitions for organ involvement were based on a combination of clinical, laboratory, or microbiological/histopathological criteria.
